# Supplementary material for: Iterative improvement in the automatic modular design of robot swarms
Source: PeerJ Comput Sci. 2020 Dec 7;6:e322. doi: 10.7717/peerj-cs.322 (PMC7924708; doi:10.7717/peerj-cs.322)
Supplement: Supplemental Information 3 [file peerj-cs-06-322-s003.zip › argos3/doc/api/standalone/a00433_source.html]

ARGoS: plugins/robots/eye-bot/simulator/qtopengl\_eyebot.h Source File


- Main Page
- Related Pages
- Namespaces
- Classes
- Files

- File List
- File Members

# plugins/robots/eye-bot/simulator/qtopengl\_eyebot.h

Go to the documentation of this file.

```
00001 
00007 #ifndef QTOPENGL_EYEBOT_H
00008 #define QTOPENGL_EYEBOT_H
00009 
00010 namespace argos {
00011    class CQTOpenGLEyeBot;
00012    class CEyeBotEntity;
00013 }
00014 
00015 
00016 #ifdef __APPLE__
00017 #include <gl.h>
00018 #else
00019 #include <GL/gl.h>
00020 #endif
00021 
00022 namespace argos {
00023 
00024    class CQTOpenGLEyeBot {
00025 
00026    public:
00027 
00028       CQTOpenGLEyeBot();
00029 
00030       virtual ~CQTOpenGLEyeBot();
00031 
00032       virtual void Draw(CEyeBotEntity& c_entity);
00033 
00034    protected:
00035 
00037       void SetLEDMaterial(GLfloat f_red, GLfloat f_green, GLfloat f_blue);
00038 
00040       void SetBodyMaterial();
00041 
00043       void MakeLeg();
00044 
00046       void DrawSideLED();
00047 
00049       void DrawBottomLED();
00050 
00052       void DrawBody();
00053 
00054    private:
00055 
00057       GLuint m_unLists;
00058 
00060       GLuint m_unLegList;
00061 
00063       GLuint m_unBodyList;
00064 
00066       GLuint m_unSideLEDList;
00067 
00069       GLuint m_unBottomLEDList;
00070 
00072       GLuint m_unVertices;
00073 
00074       /* Angle gap between two leds */
00075       GLfloat m_fLEDAngleSlice;
00076 
00077    };
00078 
00079 }
00080 
00081 #endif
```

---

Generated on 10 Jul 2018 for ARGoS by 
 1.6.1
